# Detection of Colorectal Cancer (CRC) by Urinary Volatile Organic Compound Analysis

**DOI:** 10.1371/journal.pone.0108750

**Published:** 2014-09-30

**Authors:** Ramesh P. Arasaradnam, Michael J. McFarlane, Courtenay Ryan-Fisher, Erik Westenbrink, Paula Hodges, Matthew G. Thomas, Samantha Chambers, Nicola O'Connell, Catherine Bailey, Christopher Harmston, Chuka U. Nwokolo, Karna D. Bardhan, James A. Covington

**Affiliations:** 1 Clinical Sciences Research Institute, University of Warwick, Coventry, Warwickshire, United Kingdom; 2 School of Engineering, University of Warwick, Coventry, Warwickshire, United Kingdom; 3 Department of Gastroenterology, University Hospital Coventry & Warwickshire, Coventry, Warwickshire, United Kingdom; 4 Department of Gastroenterology, Rotherham General Hospital, Rotherham, Yorkshire, United Kingdom; 5 Department of Surgery, University Hospital Coventry and Warwickshire, Coventry, Warwickshire, United Kingdom; 6 MOAC Doctoral Training Centre, University of Warwick, Coventry, Warwickshire, United Kingdom; The University of Texas MD Anderson Cancer Center, United States of America

## Abstract

Colorectal cancer (CRC) is a leading cause of cancer related death in Europe and the USA. There is no universally accepted effective non-invasive screening test for CRC. Guaiac based faecal occult blood (gFOB) testing has largely been superseded by Faecal Immunochemical testing (FIT), but sensitivity still remains poor. The uptake of population based FOBt testing in the UK is also low at around 50%. The detection of volatile organic compounds (VOCs) signature(s) for many cancer subtypes is receiving increasing interest using a variety of gas phase analytical instruments. One such example is FAIMS (Field Asymmetric Ion Mobility Spectrometer). FAIMS is able to identify Inflammatory Bowel disease (IBD) patients by analysing shifts in VOCs patterns in both urine and faeces. This study extends this concept to determine whether CRC patients can be identified through non-invasive analysis of urine, using FAIMS. 133 patients were recruited; 83 CRC patients and 50 healthy controls. Urine was collected at the time of CRC diagnosis and headspace analysis undertaken using a FAIMS instrument (Owlstone, Lonestar, UK). Data was processed using Fisher Discriminant Analysis (FDA) after feature extraction from the raw data. FAIMS analyses demonstrated that the VOC profiles of CRC patients were tightly clustered and could be distinguished from healthy controls. Sensitivity and specificity for CRC detection with FAIMS were 88% and 60% respectively. This study suggests that VOC signatures emanating from urine can be detected in patients with CRC using ion mobility spectroscopy technology (FAIMS) with potential as a novel screening tool.

## Introduction

Colorectal cancer (CRC) is one of the leading causes of cancer related death in Europe and the USA [1], [2]. At present there is a lack of effective, non-invasive screening tests for CRC. Current methods utilise guaiac based faecal occult blood (gFOB) testing, however, this has now largely been replaced by Faecal Immunochemical Testing (FIT). Whilst this is an improvement, FIT still shows relatively low sensitivity for CRC, 66–88%, depending on the cut off values for haemoglobin (50–200 ng/ml), with a specificity of 87–96% [3]–[5]. The sensitivity for advanced adenoma is even lower at 27–41%, with a specificity of 91–97% [5]. The uptake of screening utilising faecal samples is also an issue, with approximately 50% of invited participants not accepting population based FOBt screening in our locality.

Non-invasive testing of cancers, using Volatile Organic Compounds (VOCs) and gases that emanate from urine, breath, stool and blood, has received growing interest and has been an expanding area of research in recent years. This work initially started from the use of canines to detect cancers, which showed a marked ability to discriminate cancer patients from healthy individuals [6], [7], [8]. However, more recently a number of groups have indicated that it is possible to use gas phase analytical instruments, specifically gas chromatography and mass spectrometry (GC-MS), selective-ion flow mass spectrometer (SIFT) and the electronic nose (e-nose), to detect lung, breast, bladder and prostate cancers [9], [10], [11], [12]. For a detailed review on gas phase biomarkers in Gastroenterology, please see Arasaradnam et al [13].

In direct relation to colorectal cancer (CRC), recent work has shown that it is possible to discriminate cancer from non-cancer patients, but can also be used for the discrimination of lung, breast, prostate and colorectal cancer from each other by analysing breath samples [14]. This is further supported by recent work that has also shown that CRC can be distinguished from controls with over 75% accuracy using GC-MS, again employing breath analysis [15]. VOCs present in urine have also been shown to distinguish CRC patients from control groups and other cancers (leukaemia and lymphoma), with GC-MS [16]. The electronic nose (Cyrano 320, Sensigent, USA) has also been shown to discriminate between CRC and healthy controls when the VOCs profile in faeces are analysed (85% sensitivity and 87% specificity). Additionally the e-nose was able to discriminate between advanced adenomas and healthy controls with 62% sensitivity and 86% specificity [5]. These studies support the existence of putative gas phase bio-markers within biological output media for detecting CRC and thus could be the basis of a rapid screening tool.

VOCs can exist in the gaseous phase and are present in exhaled air, sweat, urine and faeces [17], [18]. The mechanism for the generation of VOCs is the subject of current research but they are perturbed in many physiological and pathological states - affected by diet and disease states. It is believed that the generation of VOCs within the bowel are the result of colonic bacteria undergoing fermentation of non-starch polysaccharides – fibre consumed by the host. As such, they represent the complex interaction of colonic cells, human gut microflora and invading pathogens [19], [20]. The study of the resultant products of fermentation which we have termed ‘the fermentome’ [17], [18], [21], [22] can be measured in urine. The latter is presumed possible due to the altered gut permeability afforded in certain gut diseases [23]. We believe that VOCs represent a bio-signature that represents the sum of the multifactorial influences (genetics, environmental factors including diet and disease states) affecting an individual. The aim of this study was to test the potential of FAIMS – a novel highly sensitive technology to differentiate between CRC and healthy controls using only urine samples.

## Materials and Methods

### 2.1 Subjects

One hundred and thirty three individuals were recruited prospectively for this study. Eighty-three of these patients had histologically confirmed colorectal cancer and fifty were healthy individuals who had a recent normal colonoscopy. The mean age of the CRC patients was 60 years (SD 17 years), 53 (64%) were male. The demographics of the subjects are shown in Table 1.

**Table 1:** Demographic data for CRC and control patients.

*N = 133*	*CRC*	*Controls*
Number	83	50
Mean Age (years)	60 (17)	47 (16)
Sex: M/F	53∶30	21∶29
Mean BMI	27 (7)	26 (5)
Current Smokers (% of whole population)	6.0%	1.5%
Alcohol: Greater than recommended units/week (% of whole population)	5.3%	3.8%

Figures in parenthesis are standard deviations (SD).

### 2.2 Study Design

This was a case control study where patients were recruited from outpatient clinics at University Hospital Coventry & Warwickshire, UK. Urine was then collected in standard universal Sterilin specimen containers (Newport, UK) and frozen immediately to −80°C, for subsequent batch analysis.

### 2.3 Analysis

Samples were thawed at room temperature overnight, aliquoted into appropriate sample bottles and analysed using the FAIMS experimental methods described below.

#### 2.3.1 FAIMS

For FAIMS analysis, a commercial instrument was utilised (Lonestar, Owlstone, UK, employing an ATLAS sampling system and split flow box). This system achieves separation of chemical components on the basis of differences in the electric field dependence of ionised chemical mobilities. FAIMS allows gas molecules to be separated and analysed at atmospheric pressure and room temperature, unlike similar traditional analytical techniques. After a sample is ionised, it is composed of ions of various sizes and types. These are introduced between two metal plates and an asymmetric high voltage waveform is applied to these plates, subjecting the ionized molecules to high electric fields. The difference in movement of these molecules within this high electric field can be measured, thus resulting in a separation of the complex mixture.

**Figure 1:**
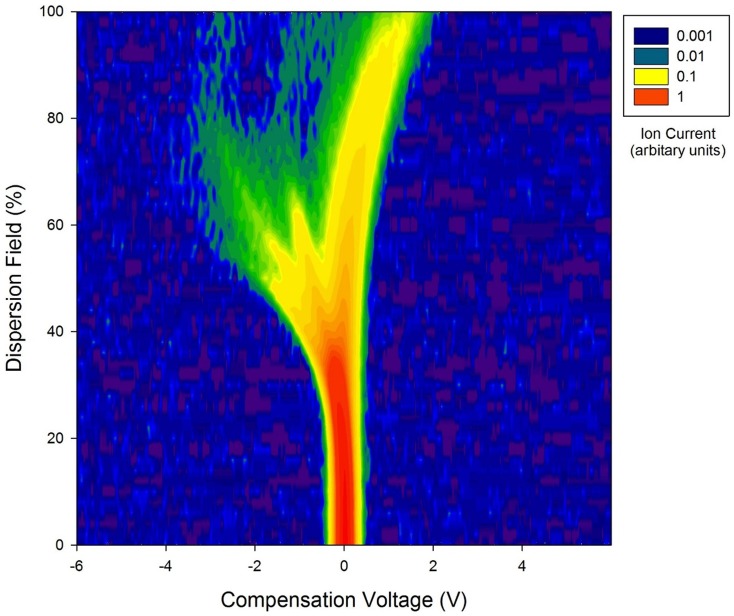
Log of raw data from the FAIMS instrument for a colorectal cancer patient. Intensity is in arbitrary units of ion count.

A sample of 5 ml of urine is aliquoted into a 20 ml glass vial and placed inside the ATLAS sampler. The sampler heats the sample to (in our case) 40±0.1°C, when the sample reaches the correct temperature (typically 10 min), clean synthetic air is passed over the sample and into the Lonestar FAIMS instrument. The flow rate over the sample is 500 ml/min and increased to 2 L/min by additional clean air before being passed into the instrument. The Lonestar is set up to scan between 0 and 90% dispersion field (the dispersion field represents the magnitude of the electric field) in 51 steps and a compensation voltage of between −6 V and +6 V in 512 steps. The compensation voltage is used to remove the effect of the drift produced by the high electric field, thus only molecules that have a specific mobility exit the plates at that point. Figure 1 shows a typical FAIMS ‘plume’ produced from a CRC patient's urine sample plotted on a log scale.

#### 2,3.2 GC-MS

For GC-MS analysis, separate aliquots of 5 ml are taken from each sample to run through a Bruker Scion SQ GC-MS instrument. Each aliquot was agitated and heated to 60°C for 5 minutes, before the contents of its headspace are extracted using a Combi-PAL ITEX automated pre-concentrator system. The volatiles contained in the ITEX were then released by heating to 250°C, and injected into the instrument at a split ratio of 1∶20 with helium carrier gas. The Restek Rxi-624Sil column (20 m length, 0.18 mm ID, 1.0 um df) fitted to the GC was kept at a constant 50°C for 1 minute before being increased up to 280°C at a rate of 20°C/min, separating out the constituent VOCs in terms of molecular weight and polarity. After an initial detection to produce a chromatogram, the VOC molecules are fragmented by the mass spectrometer and detected to produce corresponding mass spectra. These are correlated with the GC chromatogram peaks, and checked against a National Institute of Standards and Technology library (NIST 2013) of chemical compounds. This was perfrimed in order to gain additional insight into the specific chemical groups that make up the volatile headspace of the urine samples in those with CRC.

### 2.4 Statistical Methods

Data analysis for FAIMS results is performed using Fisher Discriminant Analysis. This allows for the simple interpretation of complex data to determine if differences in groups can be detected. The data was processed in Matlab (Mathworks Inc., USA, R2013b). For analysis, both the positive and negative ion count matrices of each sample were concatenated into a single 52,224 element vector (or 1D array). These were then wavelet transformed using a Daubechies D4 wavelet, a technique commonly used in data compression and has the ability to separate out subtle signals within a dataset. Data points within the 52,224 elements suitable for discrimination are then identified. This is achieved by calculating the within class scatter (Σσ_i_) and the between class scatter [(σ_μ_)^2^/(Σσ_i_)^2^] for each point within the vector (thus the same datapoints (or variable) from all the sample datasets are examined and the within class as well as between class scatter calculated across all the samples), to generate two further 1D arrays, again formed of 52,224 data points. Different thresholds are then set for within class and between class scatter and the variables that are within these thresholds are then used for data processing by fisher discriminant analysis (FDA; a pre-classified linear technique). To test the validity of the FDA, five samples from each group (CRC and controls) were removed before the FDA was performed. Then, based on the FDA results of the remnant, a prediction is made on the group of the unknown samples. This prediction is based on a KNN (k-nearest neighbor) method. This process is repeated ten times for each pair threshold values until optimum thresholds, and thus set of variables, are identified. This variable group is then used for the remainder of the analysis process to calculate sensitivity and specificity. For further details on analyses described in detail, please see Covington et al [23].

### 2.5 Ethics

Scientific and ethical approval was obtained from local Research & Development Department and Warwickshire Ethics Committee 09/H1211/38. Written informed consent was obtained from all patients who participated in the study.

## Results

The demographic data of the cancer and non-cancer group are described in Table 1. No statistically significant difference between the groups was noted however, as expected there was male gender predominance. Details of the tumour staging for the 83 CRC patients are shown in Table 2.

**Table 2:** Staging for CRC patients using the tumour, nodal and metastases classification (TNM).

*N = 83*	*T_1_*		*T_2_*		*T_3_*		*T_4_*	
	N_0_	N_1/2_	N_0_	N_1/2_	N_0_	N_1/2_	N_0_	N_1/2_
Non-metastatic (M_0_)	3	1	6	5	19	19	5	7
Metastatic (M_1_)	0	0	0	0	4	1	0	4

N.B. 9 CRC patients could not be fully staged (inoperable).

**Figure 2:**
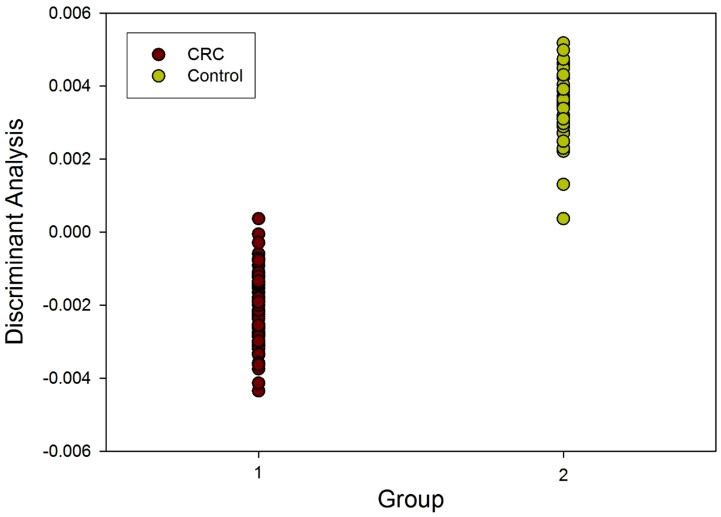
Fisher Disciminant Analysis (FDA) of FAIMS data in patients with CRC and controls.

The analysis of the FAIMS data for CRC patients and controls was carried out using Fisher Discriminant Analysis, as described above, and the results from the identified variables are shown in Figure 2. Reclassification for CRC was correct in 74% of cases (p<0.001). The sensitivity and specificity of the FAIMS analysis to detect CRC were 88% and 60% respectively.

The CRC patients' urine was also analysed by GC-MS, the results can be found in table 3, along with a list of corresponding chemicals from the National Institute for Standards and Technology (NIST) database. No unique chemical was identified in those with CRC compared with controls.

**Table 3:** GC-MS peaks for the CRC patients and corresponding National Institute of Standards and Technology (NIST) targets for these peaks.

*Average GC Retention Time (min)*	*Major MS Peaks*	*Common NIST targets*
1.4	42, 43, 45, 56, 58	Acetaldehyde, Ethylene Oxide, Oxalic Acid
1.53	42, 44, 55, 67	Dimethyl Diazene, Cyclobutyl Amine, Oxepane
1.75	42, 43, 58	Acetone
2.95	39, 43, 44, 58, 71, 86	2-Pentanone, 3-methyl-2-Butanone, 2,3-Butanedione
4.56	43, 58, 71	4-Heptanone, 3-Heptanone, 2,4-dimethyl-3-Pentanone
4.7	44, 51, 63, 78, 104	Acetyloxime-Pyridine Carboxaldehyde, Hydrocinnamoyl-Bezene-ethanamine, Styrene, Dimethyl-Thiourea
4.77	39, 40, 60, 72, 99	Allyl Isothiocyanate, Isothiocyanato-cyclopropane, 2-cyano-acetamide
5.31	42, 44, 56, 75, 94, 118, 133, 151	Methoxy-phenyl-oxime, Ethylbenzoic acid (pentyl ester), Carbamic acid (methyl ester)
5.38	41, 43, 44, 57, 72	Hexen-1-ol, 4-methyl-1-hexene, Hexanal

## Discussion

This study is the first to our knowledge to report the utility FAIMS analyses for CRC detection in urine and supports previous work by others using electronic nose [5] and GCMS [16]. This has been achieved by investigating how gases and vapours (VOCs) emanating from urine samples are disparate in those with CRC compared with controls. Our findings also support previous work where, VOC signature differences were noted in CRC patients within different biological materials (faeces, breath and urine) [5], [15], [16]. Our CRC cohort had a male sex predominance, as would be expected for a population of CRC patients, whilst our control group had a slight female predominance. This could raise the prospect of sex bias within the cohort, however, whilst the pattern of VOCs and by association, the fermentome, could theoretically be affected by sex and age, data from our previous published studies of IBD, bile acid malabsorption, pelvic cancer and coeliac disease have not shown any propensity for age and sex affecting the VOC signals [23], [24], [25], [26], [27].

De Meij et al showed that the e-nose could discriminate CRC from healthy controls with 85% sensitivity and 87% specificity, and could also distinguish advanced adenomas from healthy controls with 62% sensitivity and 86% specificity [5]. Our study has shown using urine specimens rather than faeces – with FAIMS demonstrating a sensitivity of 88% and a specificity of 60%. This has importance especially in our local population where uptake of screening is poor due to the requirement to produce faecal samples.

Ion mobility has a number of advantages over both GCMS and e-nose: for example it is undertaking a physical measurement of molecules instead of a chemical interaction (as would a traditional e-nose) and secondly, the sensitivity is much higher i.e. parts per billion to parts per trillion. This makes it an ideal platform for a future screening tool especially as the sensitivity is high. Our findings expand on previous research describing how both e-nose and FAIMS technologies can be used to distinguish effects of radiation in pelvic cancers [25] and inflammatory conditions e.g. between Crohn's disease and ulcerative colitis [26], in addition to Bile acid malabsorption and Coeliac disease [23], [27].

Genetic stool markers have received interest as a potential non-invasive screening target for CRC. Lidgard et al [28] performed a study of automated stool DNA analysis for β-actin, mutant *kRAS*, aberrantly methylated *BMP3* and *NDRG4*, and faecal haemoglobin. This showed a sensitivity of 98% and 90% specificity for CRC and 57–83% sensitivity for advanced adenomas depending on size. Our study shows comparable sensitivity results, but at a much lower process cost, and with urine rather than faecal sampling.

The unique chemical fingerprint or ‘bio-odorant fingerprint’ produced by the different disease states, and healthy individuals, shows the potential of this technology to screen for, and aid, in the diagnosis of CRC. It also has the potential to aid in further investigation of individuals with other gastrointestinal diseases. Gases and vapours are thought to be produced by the process of colonic fermentation involving a complex interaction between the colonocyte cells, human faecal flora, mucosal integrity and invading pathogens [17], [18]. VOCs emitted from bodily fluids thus have huge potential as putative biomarkers for use in the assessment of gastrointestinal diseases. Alterations in the pattern of VOCs are thought to reflect changes in the gastrointestinal environment. This suggests a possible role for gut microflora dysbiosis in the pathophysiology of CRC [29].

## Conclusions

This study has shown that the VOC signature present in the urine of patients with CRC, can be distinguished from healthy controls using FAIMS. The sensitivity and specificity of FAIMS is 88% and 60% respectively for CRC. Whilst this is lower than the gold standard of colonoscopy it is comparable with current faecal stool testing including the guaiac and immunohistochemical methods. The UK uptake for screening is low; 62%, 57% and 59% uptake in the first, second and third rounds of the national screening programme [30], and around 50% currently in the local population. One of the reasons for this is the nature of the biological sample required. Offering an alternative and less intrusive option, such as urine rather than faeces, is likely to be far more acceptable to patients, and can be incorporated into screening pathways for the future.
