# Correction: Detection of Colorectal Cancer (CRC) by Urinary Volatile Organic Compound Analysis

**DOI:** 10.1371/journal.pone.0118975

**Published:** 2015-03-05

**Authors:** The PLOS ONE Staff

The fifth author’s name is incorrect. The correct name is: Phoebe Hodges.
